# Assessment of the changes in product characteristics, total ascorbic acid, total flavonoid content, total polyphenol content and antioxidant activity of dried soursop fruit tea (*Annona muricata* L.) during product storage

**DOI:** 10.1002/fsn3.3949

**Published:** 2024-02-01

**Authors:** Yen Vy Do, Quynh Nhu Thi Le, Nguyen Huu Nghia, Ngoc Duc Vu, Nhi Thi Yen Tran, N. T. Bay, Thi Tuu Tran, Long Giang Bach, Tan Phat Dao

**Affiliations:** ^1^ Faculty of Chemical Engineering and Food Technology Nong Lam University Ho Chi Minh City Vietnam; ^2^ Institute of Applied Technology and Sustainable Development Nguyen Tat Thanh University Ho Chi Minh City Vietnam; ^3^ Faculty of Food and Environmental Engineering Nguyen Tat Thanh University Ho Chi Minh City Vietnam; ^4^ TRAVIPHA Co., Ltd. Tan Phu Dong Tien Giang Vietnam; ^5^ Department of Chemistry Soongsil University Seoul South Korea

**Keywords:** *Annona muricata*, dried soursop fruit tea, packaging materials, product preservation

## Abstract

Soursop (*Annona muricata* L.) fruit tea is a health‐beneficial product that promotes economic development and addresses the issue of excessive agricultural waste. Prolonging the shelf‐life of soursop fruit tea has been of scientific interest currently. This study evaluated the effects of three types of packaging materials of soursop fruit tea (e.g., paper, paper‐combined Polyetylen (PE), and aluminum‐combined PE) and different storage temperatures (5, 15, 30, and 45°C) on various product characteristics, total polyphenol content (TPC), total flavonoid content (TFC), total ascorbic acid (TAA), and 2,2‐diphenyl‐1‐picryl hydrazyl (DPPH)/2,2′‐azino‐bis(3‐ethylbenzothiazoline‐6‐sulfonic acid) (ABTS) free radical scavenging capacity during 4 weeks of storage. The results revealed that the sample stored in aluminum‐combined PE packaging at 30°C retained most of the product's characteristics and nutritional values. This was evidenced by the moisture content of 2.49%, TAA of 3.9 ± 1.4 mg/100 g dry weight, TPC of 12.89 ± 0.47 mgGAE/g, TFC of 0.54 ± 0.004 mgQE/g, DPPH scavenging activity of 4.06 ± 0.02 mgAA/g, and ABTS scavenging activity of 13.34 ± 0.32 mgAA/g. Additionally, the microbiological quality of the sample met the standard of TCVN 9740:2013. Overall, the study highlights the importance of packaging materials and storage temperatures to maintain the nutritional quality of soursop fruit tea. It provides valuable insights into the suitable storage conditions for preserving the quality and health‐promoting effects of this product.

## Graphical abstract

Soursop (*Annona muricata* L.) fruit tea is a healthy product that helps economic development and solves the problem of excess fresh ingredients. Prolonging the shelf‐life of soursop fruit tea is an urgent matter that needs attention currently.

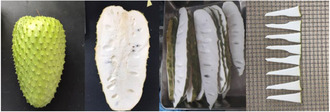

## 1 INTRODUCTION

Soursop (*Annona muricata* L.) is a tropical fruit belonging to the genus *Annona*, family Annonaceae, and native to America and Caribbean (Audu et al., 2019). In Vietnam, soursop is grown mainly in the Southern area due to favorable climate and soil conditions, particularly in Tien Giang province. According to the statistics report from Tien Giang Agriculture Industry in 2018, Tan Phu Dong district alone had more than 1108 ha of arable land with a yield exceeding 10,354 tons/year. This nutrient‐rich fruit contains a variety of vitamins and amino acids, especially ascorbic acid (approximately 29.6 mg/100 g dry matter), proline, and c‐aminobutyric acid, which can be comparable to bananas, pears, apples, grapes, and pineapple (Afzaal et al., 2022; Morton, 1987). Soursop also harbors bioactive compounds, including phenolic, flavonoids, carotene, saponins, and acetogenin have been identified in soursop fruit. For example, phenolic and carotenoids promote health benefits due to their free radicals scavenging activity, while acetogenin inhibits toxic compounds in cancer cells (Jacobo‐Herrera et al., 2019). Furthermore, soursop extracts can protect the liver through physiological mechanisms. The alkaloid components in soursop, particularly anonaine and asimilobine, are responsible for the effective anxiolytic and anti‐stress properties (Nguyen et al., 2023). The expression of apoptosis and oxidative stress‐related behaviors that are linked to diabetes are significantly decreased upon consumption of soursop (Alsenosy et al., 2019). The drying technology is mainly applied in a wide range of products such as soursop jam, soursop tea, or spray‐dried powder to reduce the product's moisture content, thereby preserving and improving the fruit nutritional values (Tan et al., 2020). In particular, tea is a popular drink all over the world. Due to the presence of antioxidants, as well as desirable taste and aroma, consumption of tea at a proper dose and frequency is recommended to promote health and prevent several chronic diseases. The production of soursop tea products has the potential for economic growth. Due to the high demand for tea, especially during national holidays and events, Vietnam is the fifth exporter for tea in the world. Currently, tea derived from local fruits is gaining popularity among young people. Soursop fruit tea is a rich source of antioxidants and nutrients to replenish energy, provide vitamins, and enhance the body's immunity (Nguyen et al., 2023). However, to prevent the loss of active ingredients in the products during processing, an appropriate drying method is required.

Drying is commonly used to remove water, minimize microbial contamination and composition degradation of raw materials during storage. Some common drying methods include freeze‐drying, convection drying, vacuum drying, heat pump drying, and infrared drying (Aktaş et al., 2017). Depending on different types of food and economic development, an appropriate drying method is selected for the product to achieve the highest yield and quality while saving energy and production cost. Heat drying is one of the most widely used food drying methods (Gunathilake et al., 2018). Previous studies have shown that the application of heat pump drying method can effectively reduce the loss of heat‐stable natural compounds (Babu et al., 2018). For tea products, after the drying process, roasting stage is necessary to enhance the unique scent, reduce the moisture content and prolong the storage time of the product. However, the roasting process affects the tea chemical composition and aroma, which mainly involved fatty acid‐derived compounds, terpenoids, phenylpropanoids/benzenoids, carotene derivatives, glycoside hydrolysis, and Maillard reaction products. The Maillard reaction, in which sugars react with amino acids to produce brown pigments with a characteristic roasted aroma and a high proportion of products (e.g., pyrazines, pyrroles, furans, and their derivatives), is one of the most important reactions in roasting (Zhu et al., 2021). For example, the study by Cao et al. (2021) on the organoleptic characteristics and chemical composition of oolong tea has shown that after roasting at 130°C, the content of flavonoids, glycosides, and procyanidins was reduced, while that of l‐theanine‐flavna‐3‐ols was increased (Cao et al., 2021).

Drying and storage are the most integral stages of post‐harvest activities, as these processes directly affect the physical and chemical properties of the product (Gamage et al., 2021). With a wide range of health benefits, it is important to ensure the quality of soursop fruit tea during storage. A study on storage of custard apple powder in bags made from different materials showed that the color difference (∆*E*) increased with prolonged storage time (Gamage et al., 2021). Furthermore, the vacuumed laminated aluminum foil pouches and polyethylene bags with vacuum exhibited higher average total ascorbic acid (TAA) and total sugar content with minimal color change than other packaging without vacuum. In addition, the vacuumed laminated aluminum foil packaging was able to preserve the product for 90 days without noticeable loss of quality (Khodifad et al., 2018). Numerous studies have reported that the product quality, color, content of ascorbic acid, lycopene, and total flavonoid content (TFC) were proportional to the storage temperature (Khodifad et al., 2018).

Indeed, the selection of appropriate packaging and storage conditions is considered essential for prolonging the product shelf‐life while maintaining their nutritional values. To the best of the authors' knowledge, the effect of packaging material and storage temperature on soursop fruit tea was limitedly known. Therefore, this study produced soursop fruit tea and evaluated the effects of storage temperature and packaging material on the moisture content, water activity, color, and nutritional properties, including TAA content, TFC, total polyphenols content (TPC) and antioxidant activity of soursop fruit tea, thereby determining the suitable conditions for effective food preservation. This study provides important insights for the food industry with an attempt to improve the productivity and limit the loss of bioactive compounds during storage.

## 2 MATERIALS AND METHODS

### 2.1 Materials

**FIGURE 1:**
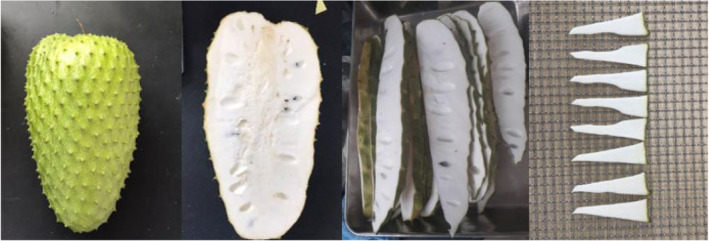
Cutting and shaping process.

Healthy three‐month‐old soursop fruit (*A. muricata* L.) with minimum brix level of 9.5°Bx was collected in Tien Giang province, Vietnam (10.4493° N, 106.3421° E) and transported to the laboratory within 8 h. The collected soursop fruits were thoroughly washed with tap water to remove dirt, microorganisms, and residual pesticides, then removed the peel and seeds, and cut vertically to a thickness of about 0.1–0.2 cm (Figure 1).

Chemicals: Na_2_CO_3_ 99.8%, AlCl_3_ · 6H_2_O 97%, HCl 36%, 2,2‐diphenyl‐1‐picryl hydrazyl (DPPH) 97%, 2,2′‐azino‐bis(3‐ethylbenzothiazoline‐6‐sulfonic acid) (ABTS) 98% and ascorbic acid 99.7% were purchased from Xilong (China), CH_3_COOK 98% from JHD (China), 2,6‐dichlorophenolindophenol (DCPIP) 99.7% from Cool Chemical (China), Folin–Ciocalteu reagent (FCR) 99%, Plate Count Agar (PCA) and Dichloran Rose Bengal Chloramphenicol (DRBC) Agarfrom Merck (Germany), and ethanol 96% (Vietnam). All reagents and chemicals were of analytical grade.

### 2.2 Production process of soursop fruit tea and experimental setup

#### 2.2.1 Production process of soursop fruit tea products

The soursop fruit tea products were produced as previously described by Nguyen et al. (2023) and Cao et al. (2021). with few modifications. Briefly, the samples were prepared by drying with heat pump at 25°C and fan speed of 50 Hz to less than 20% moisture content, followed by roasting at 130°C for 75 min until the moisture content was below 7%. The product then was cooled down at room temperature and subjected to packaging.

#### 2.2.2 Experimental setup

*Experiment 1*: Soursop fruit tea was packed with paper, paper‐combined PE, and aluminum‐combined PE (50 g per package). The samples were stored at 30°C for 4 weeks.

*Experiment 2*: Using the packaging material selected from Experiment 1. Samples were stored at 5, 15, 30, and 45°C for 4 weeks.

The color, moisture content, TAA, TFC, TPC content, the antioxidant capacity of DPPH/ABTS, and microbiological quality of soursop fruit tea samples were evaluated for 4 weeks.

**FIGURE 2:**
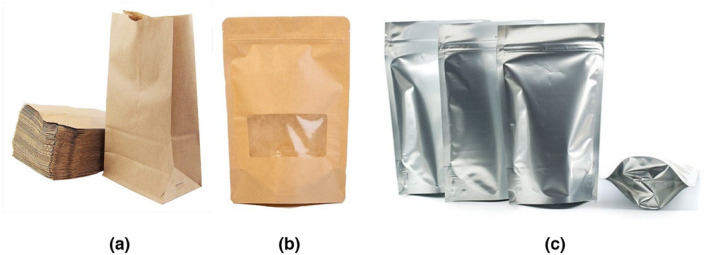
Paper packaging (a), paper combined PE packaging (b), and aluminum‐combined PE packaging (c) used in the study.

The following packaging options are available (Figure 2):

The paper packaging (P): Size of 16.5 × 21 cm, thickness of 120 μm, made from kraft paper, light brown color, and no bleaching and chemicals.
The paper combined PE packaging (P/PE): Size 15 × 22 cm, thickness 170 μm, comprises two layers of PE film laminated paper.
Aluminum‐combined PE packaging (Al): Size 15 × 22 cm, thickness 200 μm. The aluminum film was placed on the outside or in the middle of the package. At the same time, the inner layer was made from sticky material for easy packaging (e.g., PE).

### 2.3 Analytical methods

#### 2.3.1 Determination of color

Color (CIE Lab* color space) was measured using the Chromameter Konica Minolta CR‐400. The values of L*, a*, and b* were taken randomly at three positions of the fruit, then were used to calculate the color difference (Δ*E*) and browning index (BI) to determine the sample's divergence from the original sample's color, using the Equations (1) and (2) below (Abdollahzadeh et al., 2023; Ding & Ling, 2014):

(1) $$∆E=\sqrt{{\left(L^{*}-L_{0}^{*}\right)}^{2}+{\left(a^{*}-a_{0}^{*}\right)}^{2}+{\left(b^{*}-b_{0}^{*}\right)}^{2}}$$

(2) $$\mathrm{BI}=\frac{100\times \left(x-0.31\right)}{0.17},\text{where}\;x=\frac{a^{*}+1.75L^{*}}{5.645L^{*}+a^{*}-3.012b^{*}}$$

#### 2.3.2 Moisture content

The moisture content of the samples was determined by the drying method of Degnon et al. (2013) and Thao et al. (2023). The dish was dried in an oven, cooled down in a desiccator, and weighed. Then, 5 g of the sample, and the total mass of the dish and sample were weighed. The piece was then transferred to an oven at 105°C for drying until a constant mass was obtained and the mass loss was determined.

#### 2.3.3 Water activity

The water activity (*a*_w_) is an important factor in determining the quality and shelf‐life of dried foods. The *a*_w_ was determined as follows (Aktaş et al., 2017):

(3) $$a_{w}=\frac{p}{p_{0}}$$

#### 2.3.4 Determination of TAA content

The TAA content in the soursop fruit tea samples was determined based on the DCPIP titration method as previously described by Dao et al. (2022) and Manas (2014). The reaction in this solution is optimal at pH 3–4 and indicated by pink color, based on the oxidation of ascorbic acid with DCPIP acid into dehydroascorbic acid and colorless derivatives. The TAA content was calculated based on Equation (4):

(4) $$\mathrm{TAA}\;\left(\mathrm{mg}/g\right)=\frac{\left(V_{0}-0.05\right)\times V_{\mathrm{dm}}}{10\times m_{m}}\times \frac{m_{c}}{\left(V_{C}-0.05\right)},$$

where $V_{0}$ and $V_{C}$ are the sample and standard titrated DCPIP volume (mL), respectively; $V_{\mathrm{dm}}$ is the volume of volumetric flask (mL); $m_{c}$ and $m_{m}$ are the weight of ascorbic acid and sample, respectively (g).

#### 2.3.5 Determination of TPC

Total polyphenol content of soursop fruit tea extract was determined by the Folin–Ciocalteu colorimetric method, using gallic acid as a standard and previously described by Gyesi et al. (2019) and Dao et al. (2021). A total of 1 g of soursop fruit tea was weighed, homogenized with 50 mL of ethanol, and filtered to obtain the extract. The extract (0.1 mL) was placed in a dark‐colored test tube and added with 0.5 mL of FCR 10%, and 0.4 mL 7.5% Na_2_SO_3_. The sample was incubated in the dark for 1 h before absorbance measurement at the wavelength of 765 nm using GENESYS™ 10S UV–Vis spectrometer (Thermo Scientific, USA). TPC is expressed in milligrams of Gallic acid equivalent (GAE) and calculated by Equation (5).

(5) $$\mathrm{TPC}=\frac{C_{x}\times n\times V\times 100}{m\times \left(100-X\right)}\times 10^{-3},$$

where $C_{x}$ is the concentration of gallic acid determined from the standard curve (μg/mL): $C_{x}=61.0676\times A+1.8598$, *R* = 0.9991; n is the dilution from the original extract; V is the volume of the original extract (mL); *X* is the sample moisture (%) and *m* is the weight of sample (g).

#### 2.3.6 Determination of TFC

The TFC of the soursop fruit tea was determined by aluminum chloride colorimetric assay (Bryan‐Thomas, 2016; Murungweni et al., 2023). A total of 1 g of soursop fruit tea was weighed, homogenized with 50 mL of ethanol, and filtered to obtain the extract. The extract (0.5 mL) was placed in a test tube and mixed with 4.3 mL ethanol, 0.1 mL AlCl_3_ 10%, and 0.1 mL CH_3_COOK 1 M. The sample was incubated for 30 min and then measured the absorbance by using Thermo Scientific™ GENESYS 10S UV–Vis Spectrophotometer at a wavelength of 415 nm. The TFC is expressed in milligrams of quercetin equivalent (QE) and calculated by Equation (6).

(6) $$\mathrm{TFC}=\frac{C_{x}\times n\times V\times 100}{m\times \left(100-X\right)}\times 10^{-3},$$

where $C_{x}$ is the concentration of quercetin determined from the standard curve (μg/mL): $C_{x}=176.0668\times A+1.7725$, *R* = 0.999; n is the dilution from the original extract; V is the volume of the original extract (mL); X is the sample moisture (%); and m is the weight of sample (g).

#### 2.3.7 Determination of scavenging activity using DPPH/ABTS

The antioxidant activity was evaluated in vitro by DPPH scavenging method. A total of 1 g of soursop fruit tea was weighed, homogenized with 50 mL of ethanol, and filtered to obtain the extract. The extract (0.5 mL) was placed in a test tube and added with 1.5 mL of DPPH. The sample was incubated in darkness for 30 min before optical absorbance measurement using Thermo Scientific GENESYS 10S UV–Vis Spectrometer at a wavelength of 517 nm (Brand‐Williams et al., 1995; Tran et al., 2023).

The antioxidant activity was evaluated in vitro by the ABTS scavenging method as previously described by Nguyen et al. (2023). A total of 1 g of soursop fruit tea was weighed, homogenized with 50 mL of ethanol, and filtered to obtain the extract. The extract (0.5 mL) was mixed with 1.5 mL of ABTS in a test tube. The sample was incubated in the dark for 30 min before measurement by using Thermo Scientific™ GENESYS 10S UV–Vis Spectrometer at a wavelength of 734 nm.

#### 2.3.8 Quantification of total aerobic microorganisms and yeasts, molds

The total aerobic microorganisms in soursop tea were determined by the pour plate method and dilution and plating technique according to TCVN 4884:2005 with minor modifications. Under identical conditions, the decimal dilutions of the samples were inoculated onto PCA medium, then incubated aerobically at 30°C for 72 ± 6 h. The number of microorganisms per 1 g of the sample is calculated based on the colony count obtained from each plate.

Quantification of live yeasts and molds in products was performed using dilution and plate spreading techniques, following TCVN 8275–1:2010 with few modifications. Under the same conditions, decimal dilutions of the samples were plated on DRBC medium. After incubating the inoculated plates at 30°C for 5–7 days, the number of separate yeast and mold colonies obtained on the selected plates were counted.

### 2.4 Data analysis

The experiment was performed with three replicates and statistically analyzed by Statgraphics Centurion XV.I (Statgraphics Technologies, Inc., VA, USA). The analysis of variance (ANOVA), and LSD test were used to identify statistically significant differences between the means of the treatments. The data represent the mean ± standard deviation (SD) of three replicates and p < 0.05 was considered significant.

## 3 RESULTS AND DISCUSSION

### 3.1 Effects of storage packaging on physicochemical properties, chemical composition, and biological activity of soursop fruit tea over time

#### 3.1.1 Moisture content, water activity, BI, and color difference ∆*E*

Product packaging plays a crucial role in extending the shelf‐life of a product while preserving the content of bioactive compounds. The changes in moisture, *a*_w_, BI, ∆*E* among different packaging were recorded after 4‐week period, and results are presented in Table 1.

**TABLE 1:** Variation of moisture, water activity and color of soursop fruit tea.

Packaging material	Time (week)	Moisture (%)	*a*_w_	BI	∆*E*
CS	0	1.67 ± 0.3	0.2 ± 0.01	30.15 ± 8.09^aC^	‐
P	1	9.24 ± 0.19	0.5 ± 0.02	32.73 ± 4.42^aBC^	3.44 ± 0.6 ^aC^
	2	10.48 ± 0.21	0.51 ± 0.01	34.79 ± 2.06^aAB^	3.48 ± 0.89^aBC^
	3	12.44 ± 0.45	0.54 ± 0.01	34.98 ± 0.28^aAB^	4.28 ± 0.63^aAB^
	4	16.4 ± 0.5	0.61 ± 0.01	37.07 ± 6.1^aA^	4.92 ± 2.05^aA^
P/PE	1	2.93 ± 0.33	0.22 ± 0.01	33.52 ± 2.65^aBC^	3.6 ± 1.04^aC^
	2	3.05 ± 0.25	0.32 ± 0.01	35.45 ± 2.39^aAB^	4.88 ± 2.05^aBC^
	3	5.37 ± 0.57	0.42 ± 0.02	36.79 ± 2.82^aAB^	5.99 ± 0.88^aAB^
	4	7.12 ± 0.27	0.53 ± 0.01	36.88 ± 1.03^aA^	5.96 ± 0.62^aA^
Al	1	1.81 ± 0.16	0.23 ± 0.02	31.23 ± 2.42^bBC^	3.18 ± 0.61^aC^
	2	1.9 ± 0.18	0.27 ± 0.02	31.91 ± 0.48^bAB^	4.01 ± 1.45^aBC^
	3	3.27 ± 0.22	0.38 ± 0.04	31.95 ± 0.87^bAB^	4.34 ± 1.84^aAB^
	4	4.85 ± 0.22	0.4 ± 0.01	32.07 ± 0.38^bA^	5.34 ± 1.48^aA^

*Note*: Different letters represent the difference between treatments at the *p* < .05 significance level.

Abbreviations: Al, aluminum‐combined PE; CS, control sample; P, paper; P/PE, paper combined PE.

Results have shown that the sample packed in aluminum‐combined PE packaging exhibited the lowest moisture content and increased by only 3.18% after 4 weeks, as compared to the other packaging materials. This result was consistent with the study of Fukatsu (1978) in which aluminum combined with PE laminated film was also the most suitable for green tea packaging.

The correlation between rising water activity (*a*_w_) and elevated moisture content is influenced by both the packaging material and the pressure conditions within the container (Randelović et al., 2014). In the context of foods, the water activity of less than 0.6 is regarded as microbiological stability. Table 1 shows that, over the 4‐week experimental period, paper packaging sample had the highest *a*_w_ value, followed by the paper‐combined PE and the aluminum‐combined PE packaging samples. As the water activity of paper packaging exceeds the stable threshold, it proves unsuitable for the long‐term preservation of the product. Besides, the choice of packaging materials also impacts the color of soursop fruit tea. The prolonged storage time causes the tea to become darker than the original color, which corresponds to increasing ∆*E* from 2 to 10 and BI over time. The process of browning observed in the present study is attributed to the Maillard reaction, caramelization, and oxidation of ascorbic acid and enzymes (Chơn & Thuy, 2006).

#### 3.1.2 Variation of TAA, TPC, TFC, DPPH, and ABTS by packaging materials

**FIGURE 3:**
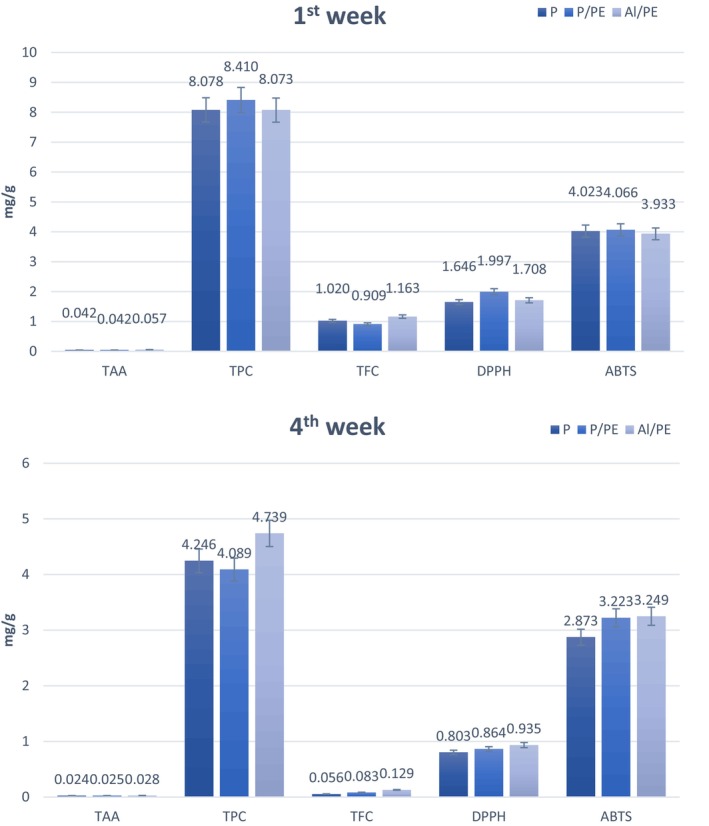
Variation of TAA, TPC, TFC, DPPH, and ABTS by packaging materials after 4 weeks of storage. Different letters represent the difference between treatments at the *p* < .05 significance level. ABTS, 2,2′‐azino‐bis(3‐ethylbenzothiazoline‐6‐sulfonic acid); DPPH, 2,2‐diphenyl‐1‐picryl hydrazyl; TAA, total ascorbic acid; TFC, total flavonoid content; TPC, total polyphenol content.

Figure 3 shows the changes in chemical composition of soursop fruit tea following a 4‐week storage period at room temperature.

The content of ascorbic acid was affected significant variability based on the packaging materials, with the highest value (64.2%) obtained in the aluminum‐combined PE packaging sample. This can be attributed to the susceptibility of TAA to environmental factors such as temperature, humidity, light, and O_2_ (Dao et al., 2022). Previously, Lee and Kader (2000) demonstrated that ascorbic acid was strongly oxidized by O_2_ during storage. In the present study, the higher permeability of paper and P/PE packaging to air, in comparison to aluminum‐combined PE packaging, resulted in elevated oxygen levels and humidity. In addition, the TPC loss rate in aluminum‐combined PE packaging was the highest at 56.6%, followed by paper packaging (55%) and P/PE packaging (49.7%). As studies have shown that l‐ascorbic acid is the main compound of TPC, the reduction of vitamin C content also associates with low TPC. Simultaneously, as the storage time prolongs, TPC tends to leach into the environment, contributing to breakdown, oxidation, and hydrolysis. However, during the second and third weeks, TPC was increased due to the presence of tannins in the soursop fruit flesh that were hydrolyzed and release sugar (usually glucose) and gallic acid (Kim et al., 2007). As shown in Figure 3, the highest rate of TFC loss was in the paper packaging sample, reaching 95.94%, followed by paper‐combined PE packaging and aluminum‐combined PE packaging with no significant difference. For paper packaging, the increase in sample moisture content during long‐term storage damaged cells and activated polyphenol oxidase enzymes, leading to the breakdown of polyphenols and flavonoids (Gamage et al., 2021; Thu et al., 2015).

The antioxidant capacity of soursop fruit tea was reduced, as evidenced by high loss rate of TPC and TFC (71%–76%). This reduction aligns with the principles of antioxidant activity in soursop fruit, as previously elucidated by Rubio‐Melgarejo et al. (2020).

#### 3.1.3 Total microorganisms of soursop fruit tea affected by the type of storage packaging over time

The total yeasts and molds of soursop fruit tea during 4 weeks of storage at room temperature were within acceptable limits (<10^4^ CFU/g according to TCVN 9740:2013), regardless the type of packaging material. Particularly, the presence of yeast and mold was observed in aluminum‐combined PE packaging at relatively low level (5 × 10^1^ CFU/g), as compared to paper packaging and paper combined PE packaging (2 × 10^2^ CFU/g) after 4 weeks of experiment.

As shown in Table 2, the total aerobic microorganisms of soursop fruit tea after 4 weeks of storage in paper packaging (1.5 × 10^4^ CFU/g) and paper combined PE packaging (1.2 × 10^4^ CFU/g) was 30 times higher than in aluminum‐combined PE packaging sample (4 × 10^2^ CFU/g), due to the rapidly increased moisture content and water activity which are favorable conditions for microorganism growth. Therefore, aluminum‐combined PE packaging was selected for microbiological quality in food.

**TABLE 2:** Microbiological test results of soursop fruit tea affected by the type of storage packaging.

Packaging material	Time (week)	Total aerobic microorganisms (CFU/g)	Total yeast, mold (CFU/g)
P	1	2.5 × 10^3^	U
	2	4.4 × 10^3^	1.5 × 10^2^
	3	4.7 × 10^3^	2.5 × 10^2^
	4	1.5 × 10^4^	3 × 10^2^
P/PE	1	1.9 × 10^2^	U
	2	5 × 10^3^	U
	3	4.5 × 10^3^	2 × 10^2^
	4	1.2 × 10^4^	2 × 10^2^
Al	1	1.1 × 10^2^	U
	2	2.7 × 10^2^	U
	3	3.6 × 10^2^	U
	4	4 × 10^2^	5 × 10^1^

Abbreviations: Al, aluminum‐combined PE; P, paper; P/PE, paper combined PE; U, undetectable.

### 3.2 Physicochemical properties, chemical composition, and biological activity over time of soursop fruit tea affected by storage temperature

#### 3.2.1 Variation of moisture content, water activity, BI, and color difference ∆*E* by storage temperature

**FIGURE 4:**
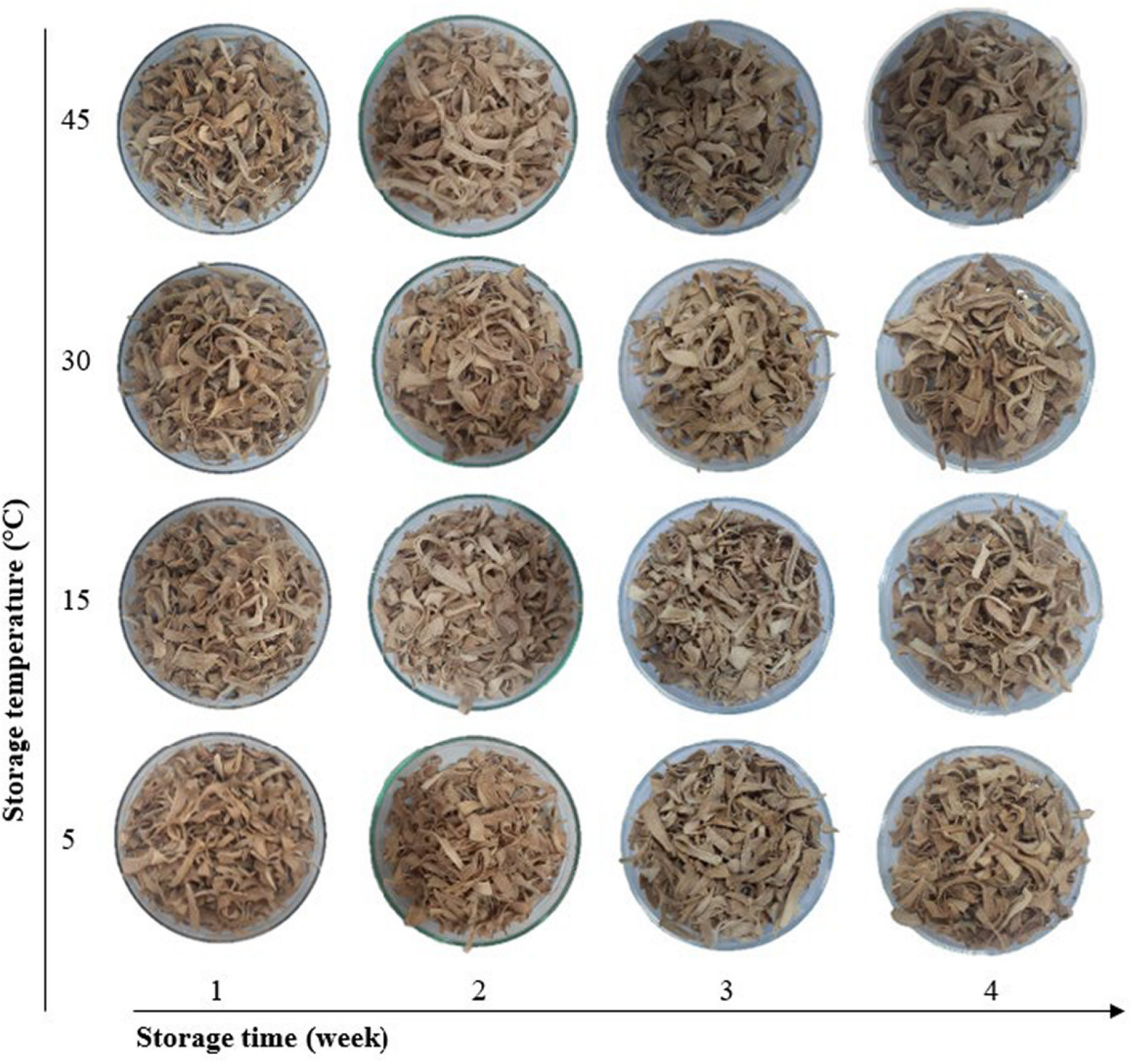
Color change of soursop fruit tea affected at different storage temperature.

Overall, the moisture and water activity of the aluminum‐combined PE packaging sample increased during storage at all investigated temperature (Figure 4; Table 3). Specifically, the moisture content at 45°C was higher than 30°C, which can be explained by the release of free water from Maillard browning reaction. Research by Miranda et al. (2014) gave similar results on samples of half of dried tomato stored at cool and room temperature. At 30°C, samples were found to have better moisture retention with less change than 5 and 45°C. However, both moisture and water activity of all tested samples remained lower than 5% and 0.6, respectively, which enhanced the color stability, inhibited the microbial growth, decelerated non‐enzymatic browning and reduced enzymatic reactions rate (Chaux‐Gutiérrez et al., 2017; Salahi et al., 2017).

**TABLE 3:** Variation of moisture, water activity and color of soursop fruit tea at different storage temperatures.

Temperature (°C)	Time (week)	Moisture (%)	*a*_w_	BI	L*	∆*E*
CS	0	0.56 ± 0.08	0.2 ± 0.001	32.04 ± 4.4	56.93 ± 1.59	‐
5	1	1.58 ± 0.19	0.252 ± 0.036	32.22 ± 1.81	55.98 ± 2.21^aA^	1.92 ± 1.7^aC^
	2	1.9 ± 0.24	0.276 ± 0.006	32.23 ± 1.56	56.90 ± 2.69^aA^	2.42 ± 0.76^aB^
	3	2.66 ± 0.31	0.285 ± 0.004	32.77 ± 2.03	55.46 ± 3.97^aA^	3.3 ± 1.87^aAB^
	4	2.96 ± 0.73	0.295 ± 0.004	33.7 ± 4.02	54.09 ± 5.01^aB^	4.69 ± 2.35^aA^
15	1	1.94 ± 0.27	0.242 ± 0.014	32.29 ± 2.49	55.88 ± 1.96^bA^	1.9 ± 1.08^aC^
	2	2.28 ± 0.4	0.256 ± 0.014	32.32 ± 1.1	53.19 ± 0.37^aA^	3.86 ± 0.36^aB^
	3	2.98 ± 0.54	0.269 ± 0.002	32.75 ± 2.08	53.29 ± 0.57^aA^	3.95 ± 0.33^aAB^
	4	4.44 ± 0.3	0.324 ± 0.006	33.54 ± 3.9	52.37 ± 1.9^bB^	4.73 ± 1.72^aA^
30	1	1.34 ± 0.23	0.208 ± 0.007	30.49 ± 1.34	56.33 ± 1.2^abA^	1.42 ± 1.1^aC^
	2	1.51 ± 0.25	0.221 ± 0.002	31.69 ± 2.42	54.61 ± 1.27^aA^	2.94 ± 0.82^aB^
	3	2.37 ± 0.29	0.233 ± 0.006	31.77 ± 3.56	54.35 ± 0.58^aA^	3.19 ± 0.68^aAB^
	4	2.49 ± 0.67	0.301 ± 0.002	31.96 ± 1.07	53.06 ± 1.58^abB^	4.31 ± 1.65^aA^
45	1	1.28 ± 0.21	0.245 ± 0.006	31.23 ± 3.32	56.73 ± 0.55^bA^	1.96 ± 1.11^aC^
	2	1.85 ± 0.48	0.250 ± 0.025	32.27 ± 1.31	53.02 ± 0.67^aA^	4.04 ± 0.66^aB^
	3	2.01 ± 0.12	0.263 ± 0.002	34.14 ± 1.55	52.8 ± 2.53^aA^	4.35 ± 2.35^aAB^
	4	3.67 ± 0.41	0.315 ± 0.005	34.35 ± 4.47	52.57 ± 1.16^bB^	4.62 ± 1.01^aA^

*Note*: Different letters represent the difference between treatments at the p < 0.05 significance level.

Abbreviation: CS, control sample.

The L* value was significantly affected by storage temperature and time (*p* < .05). The decreased L* values, as well as parameters a* and b* at all four temperature levels indicate the dark brown color according to CIE Lab* system (Table 4). The storage time factor also significantly affected ∆*E* (*p* < .05). For soursop fruit tea in aluminum‐combined PE packaging samples at all temperatures, the difference in color ∆*E* increased with storage time. The color difference was higher at 45°C than 30°C, although the difference was not significant. Due to the oxidation of ascorbic acid with storage time, dark pigments were formed, similar to the results of Chơn and Thuy (2006). Meanwhile, temperature and time did not significantly affect the BI index. At the end of four experimental weeks, the highest (34.35 ± 4.47) and the lowest BI values (31.96 ± 1.07) were obtained at 30 and 45°C, respectively.

**TABLE 4:** Microbiological test results of soursop fruit tea affected by storage temperature.

Temperature (°C)	Time (week)	Total aerobic microorganisms (CFU/g)	Total yeast, mold (CFU/g)
5	1	2.5 × 10^2^	U
	2	5.4 × 10^2^	U
	3	8.2 × 10^2^	U
	4	1.2 × 10^3^	10^2^
15	1	5 × 10^2^	U
	2	6.7 × 10^2^	U
	3	1.3 × 10^3^	5 × 10^1^
	4	1.9 × 10^3^	10^2^
30	1	10^2^	U
	2	1.6 × 10^2^	U
	3	1.8 × 10^2^	U
	4	2.3 × 10^2^	5 × 10^1^
45	1	1.2 × 10^2^	U
	2	1.5 × 10^2^	5 × 10^1^
	3	2.3 × 10^2^	10^2^
	4	3 × 10^2^	1.5 × 10^2^

Abbreviation: U, undetectable.

#### 3.2.2 Effect of storage temperature on TAA, TPC, TFC, DPPH, and ABTS

**FIGURE 5:**
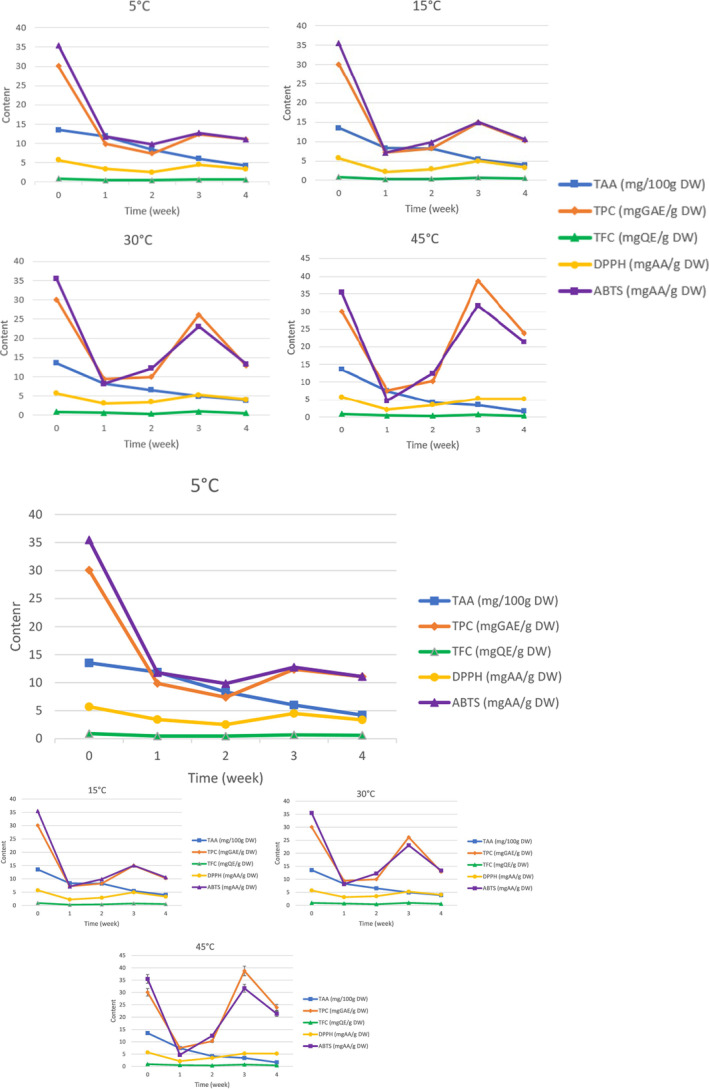
Chemical composition change graph of soursop fruit tea at 5, 15, 30, and 45°C.

Results from ANOVA statistical results showed that storage temperature substantially impacted on antioxidant capacity (*p* < .05) (Figure 5). After 4 weeks, the highest DPPH and ABTS values were recorded in samples stored at 45°C (5.2 mgAA/g DW and 21.37 mgAA/g DW, respectively), followed by 30°C (4.1 and 13.3 mgAA/g DW, respectively), 5°C (3.36 and 11.08 mgAA/g DW, respectively) and 15°C (3.31 and 10.5 mgAA/g DW, respectively). This can be explained that the low temperatures (typically 5°C) reduce the efficiency of free radical scavengers through the inactivation of antioxidants associated with refrigeration and hinder antioxidant turnover (Tripetch & Borompichaichartkul, 2019).

The present study shows that TPC was affected by storage temperature. Specifically, TPC significantly changed in response to increasing storage temperature from the second to third week, especially at 45°C (from 10.26 to 38.74 mgGAE/g DW). After 4 weeks of experiment, TPC in the samples stored at 45°C remained the highest value (23.9 mgGAE/g DW), followed by 30°C (12.88 mgGAE/g DW), 5°C (11.1 mgGAE/g DW) and 15°C (10.2 mgGAE/g DW). This can be explained by the fact that the moderate temperature (30–45°C) is favorable for the bioactive ingredients and reactions for decomposition, hydrolysis, and oxidation; increased breakdown of heat‐sensitive macromolecular compounds, enhance antioxidant capacity, especially TPC. This finding is in close agreement with previous study by Gamage et al. (2021) and Lin et al. (2016).

The temperature factor also significantly affect the degradation of TAA content (Frias et al., 2010). In the report of Md. Ayub Hossain and Klaus Gottschalk, similar results were obtained, in which the degradation rate of ascorbic acid compound in tomato pulp increased at high temperature, prolonged storage time, and high humidity (Miranda et al., 2014). The highest degradation of ascorbic acid (>80%) in soursop fruit tea samples after 4 weeks of storage was obtained at 45°C, while only about 60% of ascorbic acid was degraded at 5°C.

Results from the present study also showed that as the temperature increased from 5 to 45°C, the TFC of the investigated samples was reduced from 0.61 to 0.42 mgQE/g DW (Figure 5). This decrease in the content of physicochemical components of soursop fruit tea can be explained that when the temperature is increased, the cell membrane breaks down, releasing oxidizing and hydrolytic enzymes that can damage the antioxidants in food (Zzaman et al., 2021).

#### 3.2.3 Total microorganisms of soursop fruit tea affected by storage temperature over time

Table 4 shows that the total number of aerobic microorganisms in soursop fruit tea increased gradually with storage time. The number of microorganisms in samples at 15°C appeared on the second week, then continued to grow faster than samples at 30 and 45°C. However, at all storage temperatures, the total microorganisms were within acceptable limits (<10^4^ CFU/g according to TCVN 9740:2013). This result showed that the roasting process reduced the water activity and moisture content of the soursop samples, thus inhibiting the microorganism growth. In addition, it also confirmed that water activity significantly affects the microbial and heat tolerance in foods (Syamaladevi et al., 2016).

## 4 CONCLUSION

In the present study, aluminum‐combined PE packaging and room temperature (30°C) have been proposed as the most suitable storage conditions for soursop fruit tea to retain its nutritional content, safety, and biological activities. The results showed that after 4 weeks of storage under this condition, the remaining TAA, TPC, TFC, DPPH and ABTS scavenging activities were 28.8%, 41.9%, 45.7%, 71.1% and 37.6%, respectively, which were relatively high compared to other packaging. The microbiological quality of the products was also lower than the TCVN 9740:2013 standard. The storage conditions from the present study can be potential for production and preservation technologies of soursop fruit tea from local sources, thereby contributing to promote the nutritional values of local fruits as well as solving the environmental problem derived from agricultural waste in Vietnam.

## AUTHOR CONTRIBUTIONS

**Tan Phat Dao:** Project administration (equal); software (equal); supervision (equal); validation (equal); writing – original draft (equal); writing – review and editing (equal). **Yen Vy Do:** Investigation (equal); methodology (equal); software (equal); validation (equal); writing – original draft (equal). **Quynh Nhu Thi Le:** Investigation (equal); methodology (equal); software (equal). **Nguyen Huu Nghia:** Investigation (equal); software (equal); validation (equal). **Ngoc Duc Vu:** Methodology (equal); software (equal); validation (equal). **Nhi Thi Yen Tran:** Methodology (equal); software (equal); validation (equal); visualization (equal). **N. T. Bay:** Funding acquisition (equal); validation (equal). **Long Giang Bach:** Funding acquisition (equal); project administration (equal); resources (equal); supervision (equal); validation (equal). **Thi Tuu Tran:** Formal analysis (equal); methodology (equal); software (equal).

## CONFLICT OF INTEREST STATEMENT

The authors declare no conflict of interest.

## Data Availability

The data that support the findings of this study are available from the corresponding author upon reasonable request.
